# Prevalence and factors associated with postpartum use of long-acting reversible contraception in Bukombe District, Geita Region, Tanzania: a community- based study

**DOI:** 10.1186/s40834-020-00122-9

**Published:** 2020-12-11

**Authors:** Kiondo Solomon Kiondo, Eusebious Maro, Sophia Kiwango, Julius Pius Alloyce, Benjamin C. Shayo, Michael Johnson Mahande

**Affiliations:** 1 Kilimanjaro Christian Medical University College, Moshi, Tanzania; 2 Department of Obstetrics and Gynecology, Kilimanjaro Christian Medical Centre, Moshi, Tanzania; 3 Department of Epidemiology and Biostatistics, Institute of Public Health, Kilimanjaro Christian Medical University College, Moshi, Tanzania

**Keywords:** Long-acting reversible contraception, Postpartum, Determinants, Tanzania

## Abstract

**Background:**

Globally, approximately half of all pregnancies occur before 24 months after child birth. In Sub Saharan Africa the unmet need for family planning is highest among postpartum women. There is a dearth of information regarding factors associated with postpartum use of long acting reversible contraception (LARC) in Tanzania particularly in the Lake zone. This study aimed to determine the prevalence and factors associated with postpartum use of LARC (< 24 months) in Bukombe District, Geita Region in the Lake zone, in 2018.

**Methodology:**

Community based analytical cross-sectional study was conducted between May and June 2018 among women with less than 24 months since delivery. Multistage sampling technique was used to recruit participants. Face to face interviews with 768 postpartum women was conducted using standardized questionnaire. Data were analyzed using Stata Version 13.0. Multivariable logistic regression model was used to determine factors associated with postpartum use of LARC.

**Results:**

Prevalence of postpartum use of LARC was 10.4%. Urban residence (AOR = 2.94, 95% CI: 1.07–8.06), having formal employment (AOR = 4.81, 95% CI: 1.85–12.57) and receiving family planning counseling (AOR = 4.39, 95% CI: 1.89–10.20) were significantly associated with postpartum LARC use.

**Conclusion:**

The postpartum use of LARC was low in the studied population with implants being the most commonly used method. Urban residency, formal employment and receiving family planning counseling were associated with postpartum LARC use. Improving prenatal and quality of family planning counseling is warranted to increase postpartum LARC utilization in Bukombe.

## Introduction

Postpartum Family Planning (PPFP) is defined as the prevention of unintended pregnancy and closely spaced pregnancies through the first 12 months post-delivery [1]. Postpartum women have highest unmet need for family planning which increases their risk for mistimed and unintended pregnancies [1]. PPFP help couples to space pregnancies and achieve desired family size [1]. Postpartum contraceptives options include: short term methods such as condoms, injectables, oral contraceptives pills (OCP), long acting reversible contraception (LARC) [which includes subdermal implants and intrauterine devices (IUDs)], where as permanent methods include vasectomy and female sterilization [2].

LARC can avert early unplanned pregnancies during an extended postpartum period better than any other contraceptions [2]. LARC has been reported to be as much as 20 times more effective than oral contraceptions, transdermal patches and vaginal rings [3], with a failure rate of 1 per 100 [2]. It is most effective than any form of contraception, cost effective, long-lasting and convenient [2].

Finding fromDemographic and Health Surveys from 27 countries revealed that 95% of women within 12 months postpartum needed to prevent pregnancy in the next 24 months after birth, but majority (70%) of these women were not using contraception [4]. This reflects high unmet need for postpartum contraception among this high risk group. Findings from 21 low—and middle—income countries (LMICS) regarding pregnancy risk and postpartum contraceptive method use showed that, 61% of postpartum women who were within 24 months postpartum had an unmet need for modern contraceptives [5]. Moreover, in LMICS less than 15% of postpartum women use LARC [6] but majority (50 – 96%) of them relies on use of short acting contraceptives which are less effective in preventing unwanted pregnancies [5].

Majority (61%) of women in Tanzania have been reported to have unmet needs for family planning within 2 years postpartum, with only 31% were using the family planning methods [7]. Again, family planning method mix shows that only 10% of the women reported using highly effective methods including long acting reversible contraceptives [5] while 1% and 7% of the women of reproductive age reported using IUDs and implants respectively [8]. As a consequences of high unmet need for modern contraceptives during this period, 36% of pregnancies are unwanted [9] and 19% of all births have short birth intervals, with nearly half (47%) of all pregnancies occurring within recommended optimal interpregnancy interval [24 months] [8]. In addition Tanzania has maternal mortality ratio of 556/100,000 per live birth, which is unacceptably high [8]. Geita region has 35% unmet need for modern contraceptives among all women of reproductive age with total fertility rate (TFR) of 5.5%; these figures are above the national estimates of 22% and 5.2 for unmet need for FP and total fertility rate respectively [8].

Despite the fact that Geita region has the highest unmet need for modern contraceptives use in the country, little has been documented about factors influencing postpartum family planning use particularly long acting reversible contraceptives in this region. In addition there is paucity of data in postpartum family planning use in Tanzania, coupled with low utilization of LARC methods.

Different studies in the literature have shown predictors of postpartum LARC use such as occupation [10], secondary or tertiary education [11], high parity [12] and mode of delivery [13]. This study aimed to determine the prevalence and factors associated with postpartum use of long acting reversible contraceptives (< 24 months) in Bukombe District, Geita Region in the Lake zone.

## Methods and materials

### Study design and settings

A community-based analytical cross sectional study was conducted from May to June 2018, in Bukombe District, Geita region. Bukombe district has 3 divisions, 17 wards, 52 villages and 184 sub-villages, and it has an estimated population of 224,542. Of these, 19.1% comprise Women of reproductive age [15–49]. Bukombe district is among the districts with highest population growth rate and birth rates in Tanzania (5.9% and 4.6% respectively) [14]. The main source of income generating activities include; agriculture, animal husbandry, gold mining, petty business and formal sector employment.

The district has one hospital and two health centers which are government-owned. There are also fifteen, dispensaries where by six belong to the government and the remaining nine belongs to private sector. Only ten [10] healthcare facilities in this district provide family planning services.

### Study population, sample size and sampling techniques

This study included postpartum women [15–49 years] who were less than 24 months post-delivery from nine villages in Bukombe District. We estimated the sample size using single population proportion formula taking in consideration the following assumptions; standard deviation of 1.96 corresponding to 95% confidence interval (CI), proportion of 13 [8], marginal error of 3.5% and design effect of 2. After adding 10% for non-response rate the, estimated sample size was 780 postpartum women. However during the field work we could recruit 768 postpartum women.

Multi-stage sampling technique was used to obtain 768 postpartum women. The 1^st^ stage involved purposeful selection of 2 out of 3 divisions based on their large population size. The 2^nd^ stage involved selection of 3 wards after stratification of wards from 2 division into rural and urban, using proportion to size, 1 ward from urban settings and 2 wards from rural settings were selected. The 3^rd^ stage involved selection of 9 villages whereby in each ward 3 villages were randomly selected. At the lowest level of local administration in Bukombe district (hamlets), 3 hamlets were randomly selected in each of selected villages and postpartum mothers who were less than 24 months since delivery at the time of data collection were identified through door to door approach with the help of local leaders and those who fulfilled study inclusion criteria were invited to participate in our study.

### Study variables

The dependent variable in this study was use of long acting reversible contraceptives (Implants and IUD) during postpartum period and independent variable were socio-demographic characteristics (Age, marital status, education level, residence, occupation, income per month and Partner**’**s level of education), others were reproductive characteristics (Number of living children, mode of index delivery, place of index delivery, menstrual resumption, duration of postpartum period, counseling on FP and discussing FP use with the partner).

### Data collection methods and quality assurance

Due to the sensitive nature of this study, we used only female research assistants who were medical doctors in data collection process. All our research assistants were trained on key technical terminologies used in data collection tool as well as research ethical issues. Face to face interviews were used to collect data using structured questionnaires which was adopted from Tanzania Demographic health survey with slight modification to suit for cultural context. Information on socio-demographic characteristics, reproductive health characteristics, contraceptives use and contraceptive preference as well as children status. Principle investigator visited field sites frequently to supervise data collection activities and field staff convened with the principle investigator during the evenings to counter-check completeness of data captured before submission to data server.

### Data analysis

Data were coded, entered, cleaned and transferred to Stata Version 13.0 for the analysis purposes. Descriptive statistics were summarized using proportion for categorical data, means, standard deviation (SD) median and interquartile range (IQR) for numerical data. Multivariable logistic regression model was used to determine odds ratio and 95% confidence intervals for factors associated with postpartum LARC use. A p-value of less than 5% was considered statistically significant.

## Results

### Socio-demographic Characteristics of the participants

A total of 768 women within 24 months postpartum were invited to participate in this study, whereby all agreed and were enrolled into the study, making a 100% response rate. Two participants were excluded in the analysis due to incomplete information. The mean age of respondents was 26.9 ± 6.7 SD years. Majority, [652 (85.1%)], of participants were married, [714 (93.2%)] had primary education/below while at least two thirds were residents of rural areas. Regarding partners’ characteristics, [557 (86.2%)] of participants’ partners had primary education (Table 1).

**Table 1:** Socio-demographic Characteristics of study participants (*N* = 766)

Variables	n	%
**Age, years**		
15–24	326	42.6
25–34	318	41.5
35–49	122	15.9
**Age**^**a**^	(26.9; 6.7)	
**Marital status**		
Married	652	85.1
Unmarried	114	14.9
**Level of education**		
Primary/below	714	93.2
Secondary/above	52	6.8
**Occupation**		
Unemployed	740	96.6
Employed	26	3.4
**Estimated income per month (Tsh)**		
< 100,000	618	80.7
> 100,000	148	19.3
**Residence**		
Rural	523	68.3
Urban	243	31.7
**Partner’s level of education (n = 731)**^b^		
Primary/below	557	86.2
Secondary and above	174	23.8

Notes ^a^Mean standard deviation ^b^*n* = 731 those with partners

### Reproductive characteristics of the participants

The median number of living children was 3 (IQR: 2—5). With regard to number of living children, slightly higher than two fifth [336 (43.9%)] of the postpartum women had 1–2 children. In the current delivery, most of the participants [735 (96%)] had vaginal delivery and nearly half [371 (48.4%)] of the postpartum women had delivered at home. Nearly two-thirds [428 (58.5%)] of participants reported to discuss family planning issues with their partners, whereas, at least half of the participants [425 (55.5%)] had menstrual resumption (Tables 2 and 3).

**Table 2:** Reproductive characteristics of the study participants (*N* = 766)

Variable	n	%
**Living children**		
1–2	336	43.9
3–4	228	29.8
5 +	202	26.4
Median (IQR)	3 [2–5]	
**Mode of index delivery**		
Vaginal delivery	735	96.0
Caesarean delivery	31	4.0
**Place of index delivery**		
Home	371	48.4
Health facility	395	51.6
**Discuss FP with partner (n = 731)**^**a**^		
No	303	41.5
Yes	428	58.5
**Menstrual resumption**		
No	341	44.5
Yes	425	55.5
**Duration of postpartum period (months)**		
< 6	241	31.5
6–12	270	35.2
> 12—< 24	255	33.3
**Counseled on FP (n = 141)**^**b**^		
No	57	40.4
Yes	84	59.6

Notes: ^a^Those with partners; ^b^*n* = 141 variable limited to users only “ were you councelled on family planning methods before starting using current method”

Abbreviations: *FP* Family planning, *IQR* Interquantile range

**Table 3:** Factors associated with postpartum LARC use, Multivariable analysis (*N* = 766)

Variable	LARC use		COR (95% CI)	AOR (95% CI)	*P*-value
	**No**	**Yes n (%)**			
**Sociodemographics**					
**Age (years)**					
15–24	326	33 (10.1)	1		
25–34	318	39 (12.3)	1.24 (0.76–2.03)	0.76 (0.28–2.07)	0.592
35–49	122	8 (6.6)	0.62 (0.28–1.39)	0.40 (0.08–1.98)	0.261
**Marital status**					
Married	652	63 (9.7)	1		
Unmarried	114	17 (14.9)	1.64 (0.92- 2.92)	0.85 (0.25–2.81)	0.785
**maternal education**					
Primary/below	714	70 (9.8)	1		
Secondary/ above	52	10 (19.2)	2.19 (1.05–4.57)	0.82 (0.20–3.33)	0.784
**Occupation**					
Unemployed	740	71 (9.6)	1		
Employed	26	9 (34.6)	4.99 (2.12–11.72)	4.82 (1.85–12.57)	0.0004
**Income per month**					
< 100,000	618	65 (10.5)	1		
> 100,000	148	15 (10.1)	0.96 (0.53–1.74)	0.59 (0.22–1.57)	0.290
**Residence**					
Rural	523	32 (6.1)	1		
Urban	243	48 (19.8)	3.78 (2.32–6.15)	2.94 (1.07–8.06)	0.036
**Partner’s education (n = 731)**					
Primary/below	557	48 (8.6)	1		
Secondary and above	174	27 (15.5)	1.95 (1.17–3.24)	1.81 (0.69–4.73)	0.226
**Reproductive factors**					
**Discussing FP with partner (n = 731)**					
No	303	5 (1.7)	1		
Yes	428	70 (16.4)	11.65 (4.51–30.08)	3.23 (0.8412.41)	0.088
**Living children**					
1–2	336	36 (10.7)	1		
3–4	228	28 (12.3)	1.17 (0.69–1.97)	1.45 (0.51–4.11)	0.489
5 +	202	16 (7.9)	1.13 (0.39–1.33)	0.79 (0.20–3.03)	0.727
**Mode of index delivery**					
Vaginal delivery	735	71 (9.7)	1		
C/S delivery	31	9 (29.0)	3.82 (1.68–8.67)	2.12 (0.32–14.12)	0.435
**Place of index delivery**					
Home	371	18 (4.9)	1		
Health facility	395	62 (15.7)	3.65 (2.09–6.36)	0.59 (0.21–1.64)	0.314
**Menstrual resumption**					
No	341	16 (4.7)	1		
Yes	425	64 (15.1)	21.71 (2.02–6.41)	0.61 (0.21–1.81)	0.375
**Postpartum period**					
< 6 months	241	17 (7.1)	1		
6–12 months	270	17 (6.3)	0.89 (0.44–1.78)	0.74 (0.20–2.73)	0.654
> 12- < 24 months	255	46 (18.0)	2.9 (1.60–5.26)	0.98 (0.31–3.13)	0.973
**Counseled on FP (n = 141)**					
No	57	20 (35.16)	1		
Yes	84	60 (71.4)	4.63 (2.13–10.05)	4.39 (1.89–10.20)	0.001

*COR* Crude odds ratio, *AOR* Adjusted odds ratio, *CI* Confidence interval

### Prevalence of LARC use among postpartum women

**Fig. 1:**
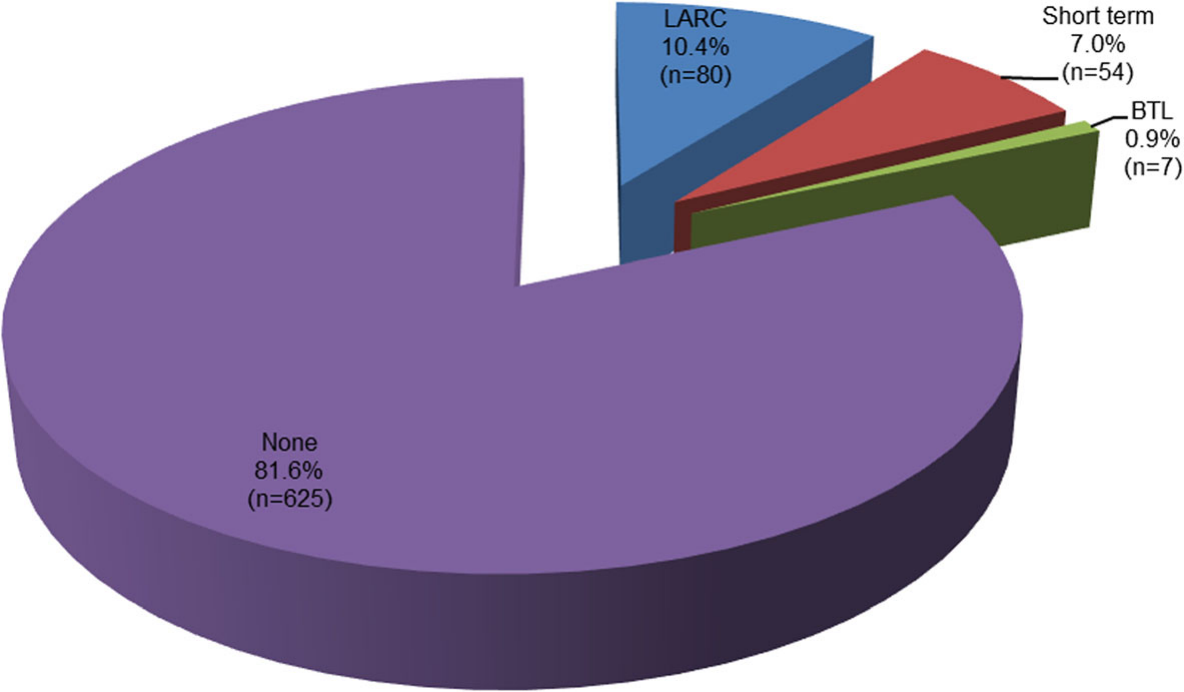
Prevalence of modern contraceptives use by method type among postpartum women

**Fig. 2:**
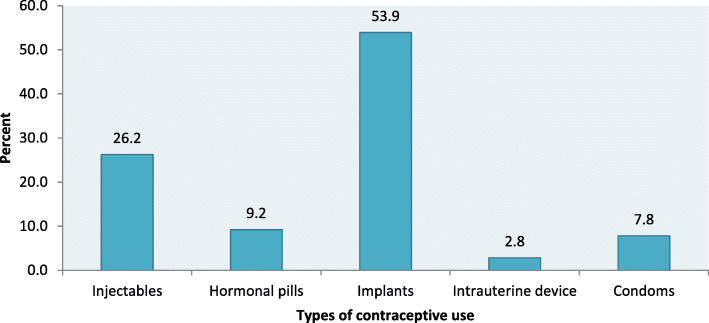
Types of Modern contraceptives used by postpartum women

Of the 766 postpartum women, 80 were using LARC, corresponding to a prevalence of 10.4%. Only eight percent reported to be using short methods, (Fig. 1). The most frequently used contraceptive methods were implants 76 (53.9%) and injectables 37 (26.2%). Other methods such as hormonal pills 13(9.2%), condoms 11(7.8%) and intrauterine devices 4 (2.8%) were infrequently used, (Fig. 2).

### Socio-demographic and reproductive characteristics associated with postpartum LARC use

Bivariate and multivariable logistic regression models were used to identify factors associated with postpartum LARC use. Bivariate analyses showed that, women with secondary education or above had two fold higher odds of LARC use compared to their counterparts with primary/below education (COR = 2.37, 95% CI:1.05–4.57), women with formal employment were almost five times more likely to use LARC compared to those who were unemployed (COR = 4.99, 95% CI: 2.12–11.72). Women who reported to reside in urban had 4-folds (COR = 3.78, (95% CI): 2.32–6.15) higher odds of using LARC compared with their rural counterparts. In addition, partners education level, partner ‘s occupation, couples discussion about FP, mode of delivery, place of delivery during the index pregnancy, menstrual resumption, postpartum duration and counseling on FP-methods were also significantly associated with postpartum LARC use.

Variables which showed significance in the bivariate analysis and those with p value less than 0.25 were selected and included in the multivariable analysis to control for possible confounders on LARC use. The multivariable logistic regression model showed that postpartum women who were residents of urban areas and those who had formal employment were almost three times more (AOR = 2.94, 95% CI: 1.07–8.06) and five times more (AOR = 4.82, 95% CI: 1.85–12.57) likely to use postpartum LARC compared to their counterparts, respectively. Furthermore, postpartum women who were counseled on FP methods were four times more (AOR = 4.39, 95% CI: 1.89–10.20) likely to use LARC than those who were not counseled about FP use.

## Discussion

In this study, we found the prevalence of postpartum use of LARC methods was as low as 10.4%. Factors such as mother’s occupation, mother’s residence and being counseled on family planning use were important predictors for postpartum use of LARC.

The prevalence of postpartum LARC use estimated in our study is consistent with the national average estimate of 10% [8]. The similarity between these findings could be explained by comparability in population characteristics studied, as majority of women prefer short methods of contraceptive than the LARC. The present finding is also in congruent with previous studies conducted within and outside Africa. For example, in a community based study in rural Uganda by Anguzu et al., the authors reported the postpartum LARC prevalence of 8.5%, [12]. Similar finding was also reported in rural China where the authors reported postpartum LARC prevalence of 9.9% [13]. In contrast, higher prevalence of postpartum LARC use (34.3%) has been reported in Ethiopia [15], Kenya (38.3%) [16] and Ethiopia (36.7%), [17]. The possible reasons for difference in findings between our study and Ethiopian studies could be due to nature of the studied population. Our population was mainly rural while participants in the Ethiopian studies were mainly from urban area where majority of women have access to information regarding the importance of FP including LARC. However, this also could be due to the fact that most of the health facilities in previous studies have high health services coverages including family planning services [17]. The difference between our study and the Kenyan counterparts could be due to the fact that, the Kenya study was a hospital based study which might provide women with better access to information regarding reproductive and sexual education including FP as compared to women in the present study whose majority (68%) were rural dwellers with poor contact with the health care providers especially those from the pastoral communities whereby nearly 50% of postpartum mothers reported to have had home delivery in during their index delivery.

In the present study, most (82%) of postpartum women were not using any form of modern contraceptives during 2 years post-delivery. Consequently this predisposes a large proportion of women in the studied communities to unplanned pregnancies, short interpregnancy intervals and their associated complications. Planning and spacing of children especially with the use of modern contraceptives especially LARC is important for the general wellbeing of the family and community at large.

In this study, majority of mothers who reported to be using LARC, were using hormonal implants. Our findings form a concordance with the national estimate which revealed that only 7% and 1% of the women of reproductive age use hormonal implants and IUDs, respectively [8]. Likewise, previous studies in Ethiopia [11, 17] and Kenya [18] showed observed that majority of women reported using hormonal implants. Findings from 12 countries in sub-Saharan African including Tanzania reported that hormonal implants were the first or second most widely used in 10 out of 12 countries studied compared with IUDs, pills and injectables [19]. The low use of IUDs could be explained by perceived misconceptions such as fear of abdomen pain, discomfort during sex and myth that IUDs can move into the abdomen during sexual intercourse [20]. Our finding is in contrast with previous study in rural China where the authors found that majority reported using IUDs than implants [13].

In this study, urban residence was an important predictor for postpartum LARC use. Postpartum women in the urban settings were more likely to use LARC as compared to their rural counterparts. This finding is consistent with previous studies conducted in northwest Ethiopia [21]. The possible explanation could be that postpartum women in urban areas have been reported to have better access to information regarding family planning and general reproductive and sexual health matters than women who reside in rural setting with poor health services [22]. This might be the case for the women in the studied population. Our findings imply that postpartum family planning services are no different, therefore there is a need for more efforts to increase utilization of modern family planning use among women in rural areas.

We also established that the occupation of the postpartum mother was associated with LARC use, particularly the formal employment. Similar relationship was reported among Kenyan women [16]. This could be explained by the fact that women with formal employment are more likely to be knowledgeable and well informed on family planning matters, have higher desire for fertility control and possibly have better access to family planning services.

Postpartum family planning counseling increased women’s likelihood of using LARC methods. Even though current study didn ‘t assess the timing of family planning counseling, but previous studies in Ethiopia [17] and Thailand [23] revealed that contraceptive counseling was associated with an increased LARC use especially when done during immediate postpartum period. Previous authors also have demonstrated that, anticipatory counseling regarding side effects and bleeding profile can help in improving acceptability and continuation rate to postpartum contraceptive use [24]. Therefore, this should be offered upon initiation of contraceptive method.

### Study limitations

The study design was a cross-section in nature which make difficult to establish the causal effect relationship. This study was done in a district where majority belong to the pastoral communities, this limit the generalization of findings whole country, but may reflect the situation in similar settings.

## Conclusion

The postpartum use of LARC was low in the studied population with implants being the most commonly used method. Postpartum women from urban residence, with formal employment, and who were counseled about LARC were more likely to use LARC.

Our finding highlights the importance of family planning counseling, particularly client should be empowered with knowledge to make informed choice, to understand common side effects of LARC and should understand the management of side effects in case needs arises. Quality counseling will help to dispel community myths and misconception towards LARC method which will help to scale up the utilization of postpartum family planning. Furthermore we call for more efforts to increase LARC use in rural areas.

## Data Availability

The datasets from this study are readily available from the corresponding author when requested.

## Abbreviations

AOR: Adjusted odds ratio
COR: Crude odds ratio
CI: Confidence Interval
CRERC: College Research Ethics and Review Committee
DELTAS: Developing Excellence in Leadership, Training and Science
IQR: Median and interquartile range
IUDs: Intrauterine devices
FP: Family Planning
LMICS: Low—and middle—income countries
LARC: Long acting reversible contraception
PPFP: Postpartum Family Planning
OCP: Oral contraceptives pills
SD: Standard deviation
